# Burden, risk factors and outcomes associated with gestational diabetes in a population-based cohort of pregnant women from North India

**DOI:** 10.1186/s12884-022-04389-5

**Published:** 2022-01-14

**Authors:** Stuti Bahl, Neeta Dhabhai, Sunita Taneja, Pratima Mittal, Rupali Dewan, Jasmine Kaur, Ritu Chaudhary, Nita Bhandari, Ranadip Chowdhury

**Affiliations:** 1 Centre for Health Research and Development, Society for Applied Studies, New Delhi, India; 2 Amrita Institute of Medical Sciences, Kochi, India; 3 Vardhman Mahavir Medical College and Safdarjung Hospital, New Delhi, India

**Keywords:** Gestational diabetes mellitus, Stillbirth, Preterm, Caesarean section, Large for gestational age

## Abstract

**Background:**

The burden of gestational diabetes mellitus (GDM) appears to be increasing in India and may be related to the double burden of malnutrition. The population-based incidence and risk factors of GDM, particularly in lower socio-economic populations, are not known. We conducted analyses on data from a population-based cohort of pregnant women in South Delhi, India, to determine the incidence of GDM, its risk factors and association with adverse pregnancy outcomes (stillbirth, preterm birth, large for gestational age babies) and need for caesarean section.

**Methods:**

We analyzed data from the intervention group of the Women and Infants Integrated Interventions for Growth Study (WINGS), an individually randomized factorial design trial. An oral glucose tolerance test (OGTT) was performed at the time of confirmation of pregnancy, and for those who had a normal test (≤140 mg), it was repeated at 24–28 and at 34–36 weeks. Logistic regression was performed to ascertain risk factors associated with GDM. Risk ratios (RR) were calculated to find association between GDM and adverse pregnancy outcomes and need for caesarean section.

**Results:**

19.2% (95% CI: 17.6 to 20.9) pregnant women who had at least one OGTT were diagnosed to have GDM. Women who had prediabetes at the time of confirmation of pregnancy had a significantly higher risk of developing GDM (RR 2.08, 95%CI 1.45 to 2.97). Other risk factors independently associated with GDM were woman’s age (adjusted OR (AOR) 1.10, 95% CI 1.06 to 1.15) and BMI (AOR 1.04, 95% CI 1.01 to 1.07). Higher maternal height was found to be protective factor for GDM (AOR 0.98, 95% CI 0.96 to 1.00). Women with GDM, received appropriate treatment did not have an increase in adverse outcomes and no increased need for caesarean section

**Conclusions:**

A substantial proportion of pregnant women from a low to mid socio-economic population in Delhi had GDM, with older age, higher BMI and pre-diabetes as important risk factors. These findings highlight the need for interventions for prevention and provision of appropriate management of GDM in antenatal programmes.

**Clinical trial registration:**

Clinical Trial Registry – India, #CTRI/2017/06/008908 (http://ctri.nic.in/Clinicaltrials/pmaindet2.php?trialid=19339&EncHid=&userName=society%20for%20applied%20studies).

**Supplementary Information:**

The online version contains supplementary material available at 10.1186/s12884-022-04389-5.

## Background

Gestational diabetes mellitus (GDM) is glucose intolerance that is first diagnosed during pregnancy most commonly at 24–28 weeks gestation, typically reverting to normal after delivery [1]. The clinical effects of GDM can range from asymptomatic to those of severe hyperglycaemia [2]. GDM poses risks for both the mother and fetus. For women with GDM, elevated glucose levels during pregnancy increases their risk of having a caesarean delivery, and the tendency to develop type 2 diabetes later in life. It also increases the infants’ risk of being born too large and developing obesity or diabetes in the future [3]. Women with GDM are also more likely to have recurrent GDM in subsequent pregnancies [4].

The estimated prevalence of GDM varies from < 1 to 28% in different countries [5]. Data from high-income countries indicate that GDM complicates up to 12.4 to 25.5% of pregnancies [6]. In India, GDM is defined as 2-h Oral Glucose Tolerance Test [OGTT] > 140 mg/dL by the Diabetes in Pregnancy Study Group, India and in National Guidelines [7] . There is wide variability in reported prevalence estimates for gestational diabetes in India, varying from 7% [8] to nearly 16% [9]. The burden of gestational diabetes appears to be increasing in India and may potentially be related to increasing prevalence of overweight or obesity [10]. There are limited data on population-based prevalence, risk factors and adverse outcomes of gestational diabetes, particularly in lower socio-economic populations.

We conducted analyses on data from a population-based cohort of urban and peri-urban low-to-mid-socioeconomic neighborhoods of South Delhi, India, to determine the incidence of gestational diabetes mellitus, its risk factors and association with adverse pregnancy outcomes (stillbirth, preterm birth, large for gestational age babies) and need for caesarean section.

## Methods

### Study design, setting and participants

We conducted this secondary analysis on data being collected as part of the Women and Infants Integrated Growth Study (WINGS). The study was conducted in urban and peri-urban low-to-mid-socioeconomic neighborhoods of South Delhi, India. A summary of the WINGS is provided below, details of methods have been previously published [11].

Briefly, eligible women aged between 18 and 30 years were identified through a door-to-door survey. Women who provided written consent to participate in the study were enrolled (first randomization; to receive pre-and peri-conception interventions or routine care and followed up until their pregnancies were confirmed, or for 18 months after enrollment. Once pregnancy was confirmed by ultrasonography, written consent was obtained (second randomization; to receive enhanced antenatal, postnatal, and early childhood care or routine antenatal, postnatal, and early childhood care) from women for further participation in the study. For the current analysis, we included pregnant women from the intervention group.

Pregnant women in the intervention group received at least 8 antenatal care visits. Body mass index (BMI) and HbA1c assessments were done at the time of confirmation of pregnancy. A one step oral glucose tolerance test was performed at the time of confirmation of pregnancy, 75 mg of anhydrous glucose was dissolved in 300 ml of water and given orally to the participant (fasting or non fasting) and 2 h later a venous blood sample was taken, and blood sugar tested. In woman who had a blood sugar level ≤ 140 mg/dl defined as normal, OGTT was repeated at 24–28 weeks and at 34–36 weeks of gestation. Women who had 2 h blood sugar value of > 140 mg/dl were identified to have GDM using national criteria [7]. They were initially managed with dietary counseling. Thereafter, a fasting (FBS) and post prandial blood sugar (PPBS) was done after 2 weeks. Women with PPBS of < 120 mg/dl were continued on dietary management and tests were repeated monthly in second trimester and fortnightly in third trimester. In women with PPBS of ≥120 mg/dl medical management was initiated with Metformin or Insulin. Referral to the diabetic clinic of our collaborating tertiary care hospital was done for uncontrolled cases.

### Definitions

GDM was defined as blood sugar > 140 mg/dL (7.8 mmol/L) 2 h after ingesting 75 g glucose orally at any time during pregnancy using Government of India guidelines [7]. Prediabetes was defined if HbA1c values ranged between 5.7 to 6.4% [12]. Stillbirth was defined if a baby was born with no signs of life at or after 28 weeks of gestation [13]. Preterm birth was defined as babies born alive before 37 completed of weeks of pregnancy. Large for gestational age (LGA) was defined as infant’s birth weight above the 90th percentile for gestational age using Intergrowth -21st Standards [14].

### Statistical analysis

Sociodemographic characteristics were reported as mean (SD), or proportions as appropriate. We calculated incidence (95% confidence interval: CI) of GDM occurring at any time during the pregnancy. We also calculated incidence of GDM based on gestational age; early abnormality those “Defined as OGTT > 140 mg/dL” based on the first trimester testing and (Late abnormality) those with normal or missing OGTT in the first trimesters but OGTT > 140 mg/dL based on the second/third-trimester testing. Univariable and multivariable logistic regressions were performed to ascertain risk factors associated with GDM. We also identified the potential risk factors for developing early and late GDM. The candidate variables were continuous (maternal age, height, years of schooling, early pregnancy (gestational age ≤ 20 weeks) BMI, HbA1c) and categorical (religion (Hindu and others), type of family (extended or joint, and nuclear), family wealth quintiles). The family wealth index was calculated for each participant by performing a principal component analysis based on all 33 assets owned by the household as done in national surveys [15]. The total scores were used to divide the population into five equal wealth quintiles: the poorest, very poor, poor, less poor, and least poor. We calculated unadjusted and adjusted risk ratios (RR) and their 95% CI for the association between GDM and adverse pregnancy outcomes (stillbirth, preterm birth, baby large for gestational age) and need for caesarean section. We also calculated unadjusted and adjusted RR between early or late GDM with adverse pregnancy outcomes including caesarean section. All **s**tatistical analyses were performed using STATA version 16 (StataCorp, College Station, TX, USA).

## Results

In this study 2294 women were followed up from preconception period till delivery. Socioeconomic and clinical characteristics of enrolled women before pregnancy are shown in Table 1. The study population was relatively young, with a mean (SD) age of 23.8 (3.1) years, about half of whom had higher than secondary school education, and with a monthly family income of about 20,000 INR (about 300 USD). Just over a third (, 34.9%) had height less than 150 cm. The mean (SD) BMI was 22.2 (4) kg/m2 and there was dual burden of malnutrition, with 18% women underweight and 22.8% women overweight or obese (Table 1). We provided the flow diagram in supplementary Fig. 1.

**Table 1:** Sociodemographic characteristics of pregnant women

Characteristics of pregnant women	No GDM ***n*** = 1814	GDM ***N*** = 430
Age (years), mean (SD)	23.5 (3.1)	24.7 (3.0)
Height (cm), mean (SD)	152.4 (5.6)	151.9 (5.8)
Height < 150 cm	614 (33.8)	162 (37.7)
Years of schooling		
None (0)	87 (4.8)	18 (4.2)
Primary (1–5)	157 (8.6)	41 (9.5)
Secondary (6–12)	672 (37.1)	146 (33.9)
Higher than secondary (> 12)	898 (49.5)	225 (52.3)
Working outside home	90 (5.0)	18 (4.2)
Early pregnancy BMI, mean (SD) Underweight (< 18.5 kg/m^2^) Overweight or obesity (≥25 kg/m^2^)	21.9 (3.9) 350 (19.3) 374 (20.6)	23.1 (4.2) 55 (12.8) 137 (31.9)
Hindu	1492 (82.3)	359 (83.5)
Wealth quintiles		
Poorest	324 (17.9)	59 (13.7)
Very Poor	308 (17.0)	68 (15.8)
Poor	360 (19.8)	91 (21.2)
Less Poor	380 (20.9)	94 (21.9)
Least Poor	442 (24.4)	118 (27.4)
Nuclear family^a^	609 (33.6)	145 (33.7)

All values are numbers (percentages) unless stated otherwise

^a^Nuclear family consists of a married couple and their dependent children

Table 2 shows the proportion of enrolled women who developed GDM and those who had prediabetes before pregnancy. 19.2% (95% CI: 17.6 to 20.9) pregnant women had GDM using national guidelines (2-h OGTT value of > 140 mg/dL). 5.6% (95% CI: 4.7 to 6.7) pregnant women were diagnosed in the first trimester and 14.9% (95% CI: 13.4 to 16.5) were diagnosed in the second or third trimester. About 0.2% women had diabetes and 2.7% had prediabetes before pregnancy. Four cases identified with pre-existing diabetes were excluded from the analysis of predictors and outcomes of GDM.

**Table 2:** Proportion of women with gestational diabetes and with prediabetes before pregnancy

Glycemic status	n (%)	95% CI
Gestational diabetes identified anytime during pregnancy	*n* = 2244*	
Defined as OGTT > 140 mg/dL1	430 (19.2)	17.6 to 20.9
Defined as OGTT > 152.9 mg/dL2	233 (10.4)	9.2 to 11.7
Gestational diabetes identified during first trimester pregnancy (Early abnormality)	*n* = 1986*	
Defined as OGTT > 140 mg/dL1	112 (5.6)	4.7 to 6.7
Defined as OGTT > 152.9 mg/dL2	69 (3.5)	2.7 to 4.4
Gestational diabetes identified during second or third trimester pregnancy (Late abnormality)	*n* = 2132*	
Defined as OGTT > 140 mg/dL1 Defined as OGTT > 152.9 mg/dL2	318 (14.9) *n* = 2175* 164 (7.5)	13.4 to 16.5 6.5 to 8.7
HbA1c status at the time of identification of pregnancy	*n* = 1854**	
< 5.7	1800 (97.1)	96.2 to 97.8
5.7 to 6.4	50 (2.7)	2.0 to 3.5
> 6.4	4 (0.2)	0.06 to 0.5

^1^definition based on national guidelines; ^2^definition based on WHO guidelines

* 50 women did not have an OGTT during pregnancy: ** 440 missing values for HbA1c

Table 3 shows the association between baseline characteristics of women with gestational diabetes (2-h OGTT > 140 mg/dL anytime during pregnancy). Higher age (adjusted odds ratio (AOR) 1.10, 95% CI 1.06 to 1.15 for each year), higher early-pregnancy BMI (AOR 1.04, 95% CI 1.01 to 1.07 for each unit) and higher HbA1c (AOR 1.73, 95% 1.23 to 2.44 for each unit) were identified as risk factors for GDM. Woman’s height was a protective factor (AOR 0.98, 95% CI 0.96 to 1.00, *p* = 0.027 for each cm) for GDM.

**Table 3:** Potential risk factors for developing gestational diabetes (2-h OGTT > 140 mg/dL anytime during pregnancy) in enrolled women

Risk factors for GDM	Unadjusted OR (95% CI)	Adjusted OR (95% CI)
Age (per 1 year)	1.13 (1.09 to 1.17); *p* < 0.001	1.10 (1.06 to 1.15); *p* < 0.001
Height (per 1 cm)	0.99 (0.97 to 1.00); *p* = 0.137	0.98 (0.96 to 1.00); *p* = 0.027
Schooling (per 1 year)	1.01 (0.98 to 1.03); *p* = 0.658	0.99 (0.96 to 1.02); *p* = 0.569
Working outside home	0.84 (0.50 to 1.40); *p* = 0.500	0.99 (0.57 to 1.72); *p* = 0.964
Nuclear family	1.01 (0.81 to 1.26); *p* = 0.953	0.97 (0.74 to 1.28); *p* = 0.837
Wealth quintile		
Poorest	Reference	Reference
Very Poor	1.21 (0.83 to 1.78); *p* = 0.323	1.16 (0.76 to 1.80); *p* = 0.491
Poor	1.39 (0.97 to 1.99); *p* = 0.074	1.31 (0.86 to 2.01); *p* = 0.204
Less Poor	1.36 (0.95 to 1.94); *p* = 0.093	1.17 (0.75 to 1.81); *p* = 0.490
Least Poor	1.47 (1.04 to 2.07); *p* = 0.029	1.34 (0.87 to 2.07); *p* = 0.187
Non-Hindu religion	0.92 (0.69 to 1.21); *p* = 0.543	0.90 (0.65 to 1.24); *p* = 0.519
Pre-pregnancy BMI (kg/m2) (per 1 unit)	1.07 (1.05 to 1.10); *p* < 0.001	1.04 (1.01 to 1.07); *p* = 0.013
HbA1c (%) at pregnancy confirmation (per 1 percentage)	2.05 (1.47 to 2.87); *p* < 0.001	1.73 (1.23 to 2.44); *p* = 0.002

Women who had prediabetes before pregnancy were at a higher risk for gestational diabetes (AOR 2.41, 95% CI 1.31 to 4.44, *p* < =0.005;)

Univariable and multivariable logistic regression was also performed to ascertain risk factors associated with GDM using WHO criteria (2-h OGTT > 153 mg/dL anytime during pregnancy) and baseline characteristics of women (Supplementary Table 1). The findings were similar to those obtained using national criteria (Supplementary Table 1). Univariable and multivariable logistic regression was also performed to ascertain risk factors associated with early and late GDM and baseline characteristics of women (Supplementary Table 2). We also assessed the risk factors for developing GDM any time during pregnancy categorizing early pregnancy BMI (normal BMI, underweight and overweight or obese) and HbA1c status (< 5.7% and > = 5.7%) at pregnancy confirmation (Supplementary Table 3). The findings were similar to Table 3.

Table 4 shows the association of GDM with adverse pregnancy outcomes in the context where management of GDM was supported by the research team. In this study, there was no significant association of GDM with stillbirth, preterm birth or LGA and caesarean section when we used OGTT > 140 mg/dL to define GDM. Similarly, we did not find an increased risk of caesarean section in pregnant women with GDM defined by WHO criteria (Supplementary Table 4). We also did not find any association between early or late GDM with adverse pregnancy outcomes and caesarean section (Supplementary Table 5).

**Table 4:** Association between gestational diabetes mellitus (2-h OGTT > 140 mg/dL) and adverse pregnancy outcomes

Outcome	No GDM n (%)	GDM n (%)	Unadjusted RR (95% CI)	Adjusted RR^a^ (95% CI)
Stillbirth	*N* = 1782 24 (1.3)	*N* = 430 3 (0.7)	0.52 (0.16 to 1.71)	0.55 (0.16 to 1.84)
Preterm birth	*N* = 1782 230 (12.9)	*N* = 430 48 (11.2)	0.86 (0.65 to 1.16)	0.77 (0.53 to 1.11)
Large for gestational age	*N* = 1627 21 (1.3)	*N* = 406 8 (2.0)	1.53 (0.68 to 3.42)	1.11 (0.49 to 2.52)
Caesarean section	*N* = 1757 519 (29.5)	*N* = 427 148 (34.7)	1.17 (1.01 to 1.36)	1.05 (0.87 to 1.26)

^a^adjusted for maternal age, height, years of schooling, early pregnancy (gestational age ≤ 20 weeks) BMI, HbA1c, religion, type of family, family wealth quintiles

## Discussion

The main findings of this study are that 19.2% of a population-based cohort of pregnant women from urban and peri-urban low-to-mid-socioeconomic neighborhoods in South Delhi, India were diagnosed with GDM. In this population there is a dual burden of malnutrition, with 18% women being underweight and 22.8% women being overweight or obese. Older age, higher pre-pregnancy BMI and higherHbA1c level at confirmation of pregnancy were identified as risk factors for GDM and higher height was a protective factor. Women with GDM, received appropriate treatment in this study and did not have an increase in adverse outcomes such as stillbirths, preterm births and large for gestational age babies but were more likely to give birth by caesarean section than women without GDM.

Previous studies have shown a wide variation in the prevalence of GDM from different states in India [8, 16–18]. The geographical differences in prevalence of gestational diabetes have been attributed to differences in mean age, BMI and socioeconomic status of pregnant women from different regions of the country [19]. Using the same cut-off (WHO, 2-h blood sugar > 153 mg/dL) for defining GDM, the prevalence of GDM in our study was almost similar (10.4%) as that in developed countries (12.5 to 25.5%) [6]. Our findings related to adverse pregnancy outcomes are similar to previous studies which have shown that treatment of GDM reduces the incidence of fetal macrosomia, mortality, birth trauma, and caesarean section deliveries [20, 21].

A major strength of our study is that it is a population-based assessment of GDM in women belonging to the low to mid socioeconomic strata, which is more representative of the average Indian population. In addition to providing the burden and risk factors of GDM, we also studied the association of this condition, when it was identified early and managed appropriately, with adverse pregnancy outcomes. The study also examined the risk associated with pre-diabetes at the time of pregnancy identification with GDM, and with adverse pregnancy outcomes. We used a single 2-h OGTT value at any time during pregnancy to detect GDM, which is feasible in a setting like India, where the pregnant women may not return after the first visit due to financial constraints, lack of accessibility of a health care centre and other reasons. Some limitations merit consideration. OGTT was carried on fasted and non-fasted patients which could result in false-positive GDM cases [22]. The high incidence of GDM may not be generalizable to other low- and middle-income countries as different criteria have been used to define GDM in countries [5].

This study has important programmatic implications. First, the high burden of GDM even in low socio-economic populations highlights the need to focus on its prevention, early detection and management in antenatal care programmes. Secondly, preventive interventions should target key risk factors including lowering the prevalence of obesity and overweight in women of reproductive age and detecting and managing pre-diabetes. Finally, early detection and appropriate management is not likely to increase the adverse pregnancy outcomes associated with GDM.

## Conclusions

A substantial proportion of pregnant women from urban and peri-urban low-to-mid-socioeconomic neighborhoods in Delhi had GDM, with older age, higher BMI and pre-diabetes as important risk factors and taller woman as a protective factor. These findings highlight the need for interventions for prevention and provision of appropriate management of GDM in antenatal programmes.

## Supplementary Information

**Additional file 1.**

## Data Availability

The Society for Applied Studies, India is a collaborator in the Healthy Birth, Growth, and Development Knowledge Integration (HBGDKi) initiative launched by the Bill & Melinda Gates Foundation. The data generated from the study will be shared with the HBGDKi repository (https://github.com/HBGDki). However, individual requests can be considered on a case-by-case basis. The request for data along with the detailed proposal describing the intended scientific question(s) to be addressed, should be submitted to Dr. Sunita Taneja (sunita.taneja@sas.org.in).

## Abbreviations

95% CI: 95% Confidence Interval
AOR: Adjusted Odds Ratio
BMI: Body mass index
GDM: Gestational diabetes mellitus
LGA: Large for gestational age
OGTT: Oral glucose tolerance test
WINGS: Women and Infants Integrated Interventions for Growth Study
WHO: World Health Organisation
