# Supplementary material for: Burden, risk factors and outcomes associated with gestational diabetes in a population-based cohort of pregnant women from North India
Source: BMC Pregnancy Childbirth. 2022 Jan 14;22:32. doi: 10.1186/s12884-022-04389-5 (PMC8759176; doi:10.1186/s12884-022-04389-5)
Supplement: Supplementary file 1 — Additional file 1. [file 12884_2022_4389_MOESM1_ESM.docx]

**Supplementary tables:**

**Table 1: Potential risk factors for developing gestational diabetes (2-hour OGTT >152.9 mg/dL anytime during pregnancy) in enrolled women**

| **Risk factors for GDM (>152.9)** | **Unadjusted OR (95% CI)** | **Adjusted OR (95% CI)** |
| --- | --- | --- |
| Age (per 1 year) | 1.09 (1.04 to 1.14); p<0.001 | 1.06 (1.01 to 1.12); p=0.026 |
| Height (per 1 cm) | 0.98 (0.96 to 1.01); p=0.205 | 0.99 (0.94 to 1.00); p=0.026 |
| Schooling (per 1 year) | 1.00 (0.97 to 1.03); p=0.891 | 0.99 (0.95 to 1.02); p=0.470 |
| Working outside home | 0.88 (0.45 to 1.70); p=0.695 | 1.07 (0.53 to 2.14); p=0.855 |
| Nuclear family | 0.99 (0.75 to 1.32); p=0.966 | 0.92 (0.65 to 1.32); p=0.658 |
| Wealth quintile  Poorest  Very Poor  Poor  Less Poor  Least Poor | Reference  1.26 (0.78 to 2.05); p=0.346  1.29 (0.81 to 2.05); p=0.278  1.11 (0.69 to 1.78); p=0.657  1.42 (0.91 to 2.20); p=0.120 | Reference  1.34 (0.77 to 2.35); p=0.303  1.34 (0.77 to 2.34); p=0.298  1.11 (0.62 to 1.99); p=0.731  1.51 (0.85 to 2.67); p=0.158 |
| Non-Hindu religion | 0.82 (0.56 to 1.19); p=0.291 | 0.78 (0.51 to 1.20); p=0.264 |
| Early pregnancy BMI (kg/m2) (per 1 unit) | 1.07 (1.04 to 1.11); p<0.001 | 1.05 (1.01 to 1.09); p=0.018 |
| HbA1c (%) at pregnancy confirmation (per 1 percentage) | 2.10 (1.37 to 3.23); p=0.001 | 1.80 (1.17 to 2.79); p=0.008 |

**Table 2a. Potential risk factors for developing gestational diabetes (2-hour OGTT >140 mg/dL during first trimester of pregnancy) in enrolled women**

| **Risk factors for GDM** | **Unadjusted OR (95% CI)** | **Adjusted OR (95% CI)** |
| --- | --- | --- |
| Age (per 1 year) | 1.11 (1.04 to 1.18); p<0.001 | 1.09 (1.01 to 1.17); p=0.026 |
| Height (per 1 cm) | 0.99 (0.95 to 1.02); p=0.400 | 0.97 (0.94 to 1.01); p=0.151 |
| Schooling (per 1 year) | 1.01 (0.96 to 1.06); p=0.640 | 1.02 (0.96 to 1.08); p=0.504 |
| Working outside home | 0.53 (0.17 to 1.71); p=0.290 | 0.59 (0.17 to 1.95); p=0.388 |
| Nuclear family | 1.33 (0.90 to 1.97); p=0.149 | 1.21 (0.74 to 1.98); p=0.445 |
| Wealth quintile  Poorest  Very Poor  Poor  Less Poor  Least Poor | Reference  0.69 (0.36 to 1.34); p=0.276  0.92 (0.50 to 1.66); p=0.765  0.60 (0.31 to 1.14); p=0.120  1.04 (0.59 to 1.81); p=0.900 | Reference  0.61 (0.28 to 1.30); p=0.200  0.93 (0.46 to 1.88); p=0.838  0.48 (0.21 to 1.09); p=0.079  1.01 (0.49 to 2.09); p=0.187 |
| Non-Hindu religion | 1.19 (0.74 to 1.91); p=0.477 | 1.21 (0.70 to 2.10); p=0.488 |
| Early-pregnancy BMI (kg/m2) (per 1 unit) | 1.04 (0.99 to 1.09); p=0.118 | 1.04 (0.99 to 1.09); p=0.203 |
| HbA1c (%) at pregnancy confirmation (per 1 percentage) | 2.51 (1.35 to 4.69); p<0.001 | 2.18 (1.17 to 4.08); p=0.014 |

**Table 2b. Potential risk factors for developing gestational diabetes (2-hour OGTT >140 mg/dL during second or third trimester of pregnancy) in enrolled women**

| **Risk factors for GDM** | **Unadjusted OR (95% CI)** | **Adjusted OR (95% CI)** |
| --- | --- | --- |
| Age (per 1 year) | 1.13 (1.09 to 1.18); p<0.001 | 1.10 (1.05 to 1.15); p<0.001 |
| Height (per 1 cm) | 0.99 (0.97 to 1.00); p=0.183 | 0.98 (0.96 to 1.00); p=0.087 |
| Schooling (per 1 year) | 1.00 (0.98 to 1.03); p=0.740 | 0.99 (0.95 to 1.02); p=0.317 |
| Working outside home | 0.95 (0.54 to 1.66); p=0.853 | 1.14 (0.63 to 2.08); p=0.661 |
| Nuclear family | 0.91 (0.70 to 1.17); p=0.458 | 0.91 (0.67 to 1.24); p=0.569 |
| Wealth quintile  Poorest  Very Poor  Poor  Less Poor  Least Poor | Reference  1.48 (0.94 to 2.31); p=0.088  1.63 (1.06 to 2.50); p=0.026  1.77 (1.17 to 2.70); p=0.007  1.68 (1.11 to 2.54); p=0.013 | Reference  1.42 (0.86 to 2.34); p=0.165  1.48 (0.90 to 2.42); p=0.121  1.50 (0.91 to 2.47); p=0.112  1.48 (0.89 to 2.46); p=0.129 |
| Non-Hindu religion | 0.82 (0.59 to 1.15); p=0.249 | 0.81 (0.55 to 1.17); p=0.256 |
| Early-pregnancy BMI (kg/m2) (per 1 unit) | 1.08 (1.05 to 1.10); p<0.001 | 1.04 (1.00 to 1.08); p=0.025 |
| HbA1c (%) at pregnancy confirmation (per 1 percentage) | 1.84 (1.27 to 2.69); p=0.001 | 1.51 (1.03 to 2.22); p=0.035 |

**3. Potential risk factors for developing gestational diabetes (2-hour OGTT >140 mg/dL anytime during pregnancy) in enrolled women stratified by early-pregnancy BMI and HbA1c (%) at pregnancy confirmation**

| **Risk factors for GDM** | **Unadjusted OR (95% CI)** | **Adjusted OR (95% CI)** |
| --- | --- | --- |
| Age (per 1 year) | 1.13 (1.09 to 1.17); p<0.001 | 1.10 (1.06 to 1.15); p<0.001 |
| Height (per 1 cm) | 0.99 (0.97 to 1.00); p=0.137 | 0.98 (0.96 to 0.99); p=0.039 |
| Schooling (per 1 year) | 1.00 (0.98 to 1.03); p=0.658 | 0.99 (0.96 to 1.02); p=0.523 |
| Working outside home | 0.84 (0.50 to 1.40); p=0.500 | 1.07 (0.62 to 1.87); p=0.801 |
| Nuclear family | 1.01 (0.81 to 1.26); p=0.953 | 0.97 (0.74 to 1.28); p=0.838 |
| Wealth quintile  Poorest  Very Poor  Poor  Less Poor  Least Poor | Reference  1.21 (0.82 to 1.78); p=0.323  1.39 (0.97 to 2.00); p=0.074  1.36 (0.95 to 1.94); p=0.093  1.47 (1.04 to 2.07); p=0.029 | Reference  1.23 (0.80 to 1.89); p=0.346  1.37 (0.90 to 2.10); p=0.141  1.18 (0.77 to 1.83); p=0.448  1.38 (0.89 to 2.13); p=0.146 |
| Non-Hindu religion | 0.92 (0.69 to 1.21); p=0.543 | 0.90 (0.65 to 1.24); p=0.524 |
| Early-pregnancy BMI  Normal BMI (18.5 to 24.9 kg/m^2^)  Underweight (<18.5 kg/m^2^)  Overweight or obesity (≥25 kg/m^2^) | Reference  0.72 (0.52 to 0.99); p=0.042  1.68 (1.32 to 2.13); p<0.001 | Reference  0.75 (0.52 to 1.07); p=0.109  1.37 (1.04 to 1.80); p=0.025 |
| HbA1c (%) at pregnancy confirmation  <5.7  >= 5.7 | Reference  2.80 (1.54 to 5.08); p=0.001 | Reference  2.41 (1.31 to 4.43); p=0.005 |

**Table 4: Association between gestational diabetes mellitus (2-hour OGTT >152.9mg/dL) and adverse pregnancy outcomes**

| **Outcome** | **No GDM**  n (%) | **GDM**  n (%) | **Unadjusted RR (95% CI)** | **Adjusted RR***  **(95% CI)** |
| --- | --- | --- | --- | --- |
| Stillbirth | N= 1977  26 (1.3) | N= 235  1 (0.4) | 0.32 (0.04 to 2.37) | 0.49 (0.07 to 3.71) |
| Large for gestational age | N= 1811  27 (1.5) | N= 222  2 (0.9) | 0.60 (0.14 to 2.52) | 0.47 (0.11 to1.99) |
| Preterm birth | N= 1977  253 (12.8) | N= 235  25 (10.6) | 0.83 (0.56 to 1.23) | 0.68 (0.42 to 1.09) |
| Caesarean section | N= 1951  592 (30.3) | N=233  75 (32.2) | 1.06 (0.87 to 1.29) | 0.87 (0.66 to 1.14) |

*adjusted for maternal age, height, years of schooling, early pregnancy (gestational age ≤20 weeks) BMI, HbA1c, religion, type of family, family wealth quintiles

**Table 5a: Association between first trimester (Early abnormality)** **gestational diabetes mellitus (2-hour OGTT >140 mg/dL) and adverse pregnancy outcomes**

| **Outcome** | **No GDM**  n (%) | **GDM**  n (%) | **Unadjusted RR (95% CI)** | | **Adjusted RR***  **(95% CI)** |
| --- | --- | --- | --- | --- | --- |
| Stillbirth | N= 1828  23 (1.3) | N=109  1 (0.9) | 0.72 (0.10 to 5.30) | 0.73 (0.10 to 5.34) | |
| Preterm birth | N= 1828  227 (12.4) | N= 109  9 (8.3) | 0.63 (0.33 to 1.20) | 0.65 (0.31 to 1.33) | |
| Large for gestational age | N= 1672  24 (1.4) | N=99  2 (2.0) | 1.41 (0.34 to 5.87) | 1.20 (0.29 to 4.97) | |
| Caesarean section | N= 1812  551 (30.4) | N= 109  33 (30.3) | 0.99 (0.74 to 1.33) | 0.93 (0.65 to 1.32) | |

*adjusted for maternal age, height, years of schooling, early pregnancy (gestational age ≤20 weeks) BMI, HbA1c, religion, type of family, family wealth quintiles

**Table 5b: Association between second or third trimester pregnancy (late abnormality)** **gestational diabetes mellitus (2-hour OGTT >140 mg/dL) and adverse pregnancy outcomes**

| **Outcome** | **No GDM**  n (%) | **GDM**  n (%) | **Unadjusted RR (95% CI)** | **Adjusted RR***  **(95% CI)** |  |
| --- | --- | --- | --- | --- | --- |
| Stillbirth | N= 1767  24 (1.4) | N=318  2 (0.6) | 0.46 (0.11 to 1.96) | 0.51 (0.12 to 2.18) | |
| Preterm birth | N= 1782  230 (12.9) | N= 319  39 (12.2) | 0.95 (0.69 to 1.30) | 0.83 (0.57 to 1.20) | |
| Large for gestational age | N= 1613  21 (1.3) | N=304  6 (2.0) | 1.52 (0.62 to 3.72) | 1.11 (0.44 to 2.82) | |
| Caesarean section | N= 1749  513 (29.3) | N= 317  114 (36.0) | 1.23 (1.04 to 1.44) | 1.08 (0.88 to 1.33) | |

*adjusted for maternal age, height, years of schooling, early pregnancy (gestational age ≤20 weeks) BMI, HbA1c, religion, type of family, family wealth quintiles

**Supplementary Figure**

**Figure 1: Flow diagram**

2294 pregnant women

2244 pregnant women had OGTT

430 pregnant women with GDM (defined as OGTT > 140 mg/dL)

1814 pregnant women with no GDM
